# Correspondence

**Published:** 1912-07

**Authors:** John H. Grant

**Affiliations:** No. 195 Madison Avenue, Albany, N. Y.


### CORRESPONDENCE

No. 195 Madison Avenue,
Albany, N. Y., May 11th, 1912.

Dr. A. L. Benedict,

Buffalo Medical Journal,

Buffalo, N. Y.

My dear Doctor:—

I always enjoy looking over the pages of your bright JOURNAL, and as an old soldier I like reading the installments of the interesting "Notes of Civil War Army Life" depicted by our lamented friend, Colonel Potter.

I keep in touch with medical affairs, have affiliated with the Albany County Medical Society from Erie County, of which I was a member from 1897.

Wishing the JOURNAL every success and with regret that circumstances took me away from Buffalo,

Truly Yours,
JOHN H. GRANT, M. D.

